# Ecological perspectives on synthetic biology: insights from microbial population biology

**DOI:** 10.3389/fmicb.2015.00143

**Published:** 2015-02-26

**Authors:** Ana E. Escalante, María Rebolleda-Gómez, Mariana Benítez, Michael Travisano

**Affiliations:** ^1^Department of Ecology, Evolution and Behavior, University of MinnesotaSt. Paul, MN, USA; ^2^Laboratorio Nacional de Ciencias de la Sostenibilidad, Departamento de Ecología de la Biodiversidad Instituto de Ecología, Universidad Nacional Autónoma de MéxicoMexico City, Mexico; ^3^Centro de Ciencias de la Complejidad, Universidad Nacional Autónoma de MéxicoMexico City, Mexico; ^4^BioTechnology Institute, University of MinnesotaSt. Paul, MN, USA

**Keywords:** spatial structure, mass action environment, cooperation, cheating, microbial consortia, synthetic ecology

## Abstract

The metabolic capabilities of microbes are the basis for many major biotechnological advances, exploiting microbial diversity by selection or engineering of single strains. However, there are limits to the advances that can be achieved with single strains, and attention has turned toward the metabolic potential of consortia and the field of synthetic ecology. The main challenge for the synthetic ecology is that consortia are frequently unstable, largely because evolution by constituent members affects their interactions, which are the basis of collective metabolic functionality. Current practices in modeling consortia largely consider interactions as fixed circuits of chemical reactions, which greatly increases their tractability. This simplification comes at the cost of essential biological realism, stripping out the ecological context in which the metabolic actions occur and the potential for evolutionary change. In other words, evolutionary stability is not engineered into the system. This realization highlights the necessity to better identify the key components that influence the stable coexistence of microorganisms. Inclusion of ecological and evolutionary principles, in addition to biophysical variables and stoichiometric modeling of metabolism, is critical for microbial consortia design. This review aims to bring ecological and evolutionary concepts to the discussion on the stability of microbial consortia. In particular, we focus on the combined effect of spatial structure (connectivity of molecules and cells within the system) and ecological interactions (reciprocal and non-reciprocal) on the persistence of microbial consortia. We discuss exemplary cases to illustrate these ideas from published studies in evolutionary biology and biotechnology. We conclude by making clear the relevance of incorporating evolutionary and ecological principles to the design of microbial consortia, as a way of achieving evolutionarily stable and sustainable systems.

## Introduction

Microbes have a long biotechnological history, since the first use of yeast and bacteria for fermentation. Modern innovation in biotechnology has harnessed the diversity of microbial metabolic capabilities, almost exclusively by selection or engineering of single strains. The single strain approach has a long record of success, such as the engineering of insulin producing *Escherichia coli* (Williams et al., 1982). Even so, in many cases single selected or engineered strains are not capable of producing the desired product. For example in engineered strains, genetic modification is limited by metabolic load and the number of exogenous elements that can be cloned and optimized in a single cell, among other problems (Glick, 1995; Brenner et al., 2008; Shong et al., 2012). A main goal of synthetic biology is to realize the potential of microbial consortia, to develop systems that can usefully generate products beyond that of single strains. Natural or engineered microbial consortia show great promise in overcoming the limitations of single strain systems, because of their capability for complex metabolic interactions (Verduzco-Luque et al., 2003; Raghoebarsing et al., 2006), and their inherent compartmentalization which prevents undesired cross-reactions and side products (Shong et al., 2012).

Microbial interactions are the primary advantage of consortia and provide its structure and function. Most microbial interactions in consortia are mediated by the use and excretion of metabolites in the form of small molecules (e.g., nutrients, chemical cues, etc.). Extraordinary efforts have been made in modeling metabolic networks to predict the type of interactions among species within microbial consortia to inform engineers in their design (Freilich et al., 2011; Harcombe et al., 2014). These modeling approaches include biophysical parameters (e.g., metabolite concentration, diffusion rates, viscosity of the media, etc.) that necessarily influence the strength of the interactions and the cellular growth of the microbial populations. Using these models provides the metabolic basis for product generation and frequently can be directly used to predict product output rates.

The main challenge for synthetic ecology is that consortia are frequently unstable, largely because evolution by constituent members affects their interactions. This leads to reduced yield and lower productivity (Field et al., 1995; Hamer, 1997; Kato et al., 2005; Kim et al., 2008), and thus far there are few examples of successful microbial consortia (recently reviewed in Sabra et al., 2010). Current practices in modeling consortia largely consider interactions as fixed circuits of chemical reactions, which greatly increases their tractability. This simplification comes at the cost of essential biological realism, stripping out the ecological context in which the metabolic actions occur and the potential for evolutionary change in biological systems. Consideration of metabolic, biophysical and trophic interactions from an ecological and evolutionary perspective are scant (Momeni et al., 2013). Because ecological and evolutionary principles are frequently not included in design, consortia *evolutionary* stability is not engineered into the system.

This realization highlights the necessity to better identify the key components that influence the stable coexistence of microorganisms, not only in terms of the biophysical variables and stoichiometric modeling of metabolism (Freilich et al., 2011), but also by inclusion of ecological principles as well as organismic and evolutionary understanding of the system into microbial consortia design (Freilich et al., 2009; Kuhn et al., 2010). Fortunately, there is a large literature on community stability from population and evolutionary biology, which includes experimental studies of microbial systems (Stewart and Levin, 1973; Lenski and Hattingh, 1986; Turner et al., 1996). In contrast to biotechnologists, evolutionary biologists have focused on identifying ecological mechanisms and selective regimes that affect community stability. Integrating results from microbial population biology is a powerful addition to engineering of microbial-mediated processes, as has been previously recognized (Goldman and Brown, 2009). Such integration has been hampered by the different technical terms, goals and perspectives of biotechnologists and evolutionary biologists.

Competition and cooperation are two of the most important ecological processes, and are frequently mediated by resources. Resource-based competition involves two or more species that consume the same resources, thereby potentially limiting one another's growth. In contrast, resource-based cooperation promotes growth and persistence, and typically involves consumption of metabolic products. It has two general forms, cascade or reciprocal. Cascade interactions involve one-way consumption, in which the metabolic products of one species are consumed by others. Reciprocal cooperation involve consumption of metabolic products by all species involved in the cooperative interaction (Freilich et al., 2011; Momeni et al., 2013; Grosskopf and Soyer, 2014). In natural communities, it is likely to find species as part of cooperative cycles (Freilich et al., 2011), and these type of interactions, we believe, may be the more stable in evolutionary terms for coexistence when designing microbial consortia.

Access to resources is directly affected by *connectivity*, the propensity for molecules and cells to flow across the system, or as some have referred to as the viscosity of the media (Momeni et al., 2013). Depending on the type of interaction among microbes, competition and the type of cooperation, we argue that spatial structure plays a key role in the stability of the consortia, affecting simultaneously cellular location (Momeni et al., 2013) and resource availability. For communities involving reciprocal cooperative interactions, spatial structure limits the potential for non-cooperative individuals (cheaters) to evolve and spread, and is therefore essential for stable coexistence. In contrast for non-reciprocal interactions, access to resources is imperative and restricting the flow of resources does not contribute to coexistence stability.

This review aims to bring ecological and evolutionary concepts to the discussion on the stability of selected or engineered microbial consortia. In particular, we present a critical review to frame our argument on the relevance that the combined effect of spatial structure and ecological interactions has on the persistence of these consortia. We discuss exemplary cases to illustrate these ideas from the evolutionary biology and biotech-engineering perspectives. We conclude the review by making clear the relevance of incorporating evolutionary and ecologically principles to the engineering approach taken to date in the “design” of microbial consortia as a way of achieving evolutionarily stable, sustainable, and productive biological systems.

## Ecological principles of coexistence derived from microbial population biology studies

Ecological and evolutionary research has demonstrated that biodiversity can emerge and be maintained when there is ecological opportunity and competitive trade-offs that allow the existence of different ecological types (Tilman, 1977; Rainey and Travisano, 1998). Microbial coexistence can be promoted and maintained, by simple changes that affect either resource availability or physical structure (Rainey et al., 2000). The physical structure of environments can promote stable coexistence of genetically distinct individuals by localizing connectivity and interactions (Amarasekare, 2003). Spatial structure can lead to locally depleted resources than can limit growth of competitors and result in a patchy environment with multiple niches and ecological opportunity (Chao and Levin, 1981; Rainey and Travisano, 1998; Greig and Travisano, 2004). However, spatial structure can also limit diversification by reducing connectivity, for example by decreasing resource availability in cases where resources are made available through facilitation (Saxer et al., 2009). We refer to the physical structure of an environment as either *mass action* or *structured*, the key difference between these two categories is their differences in connectivity, respectively high and low, which greatly affects the flow and potential for interactions of cells and molecules within the system (Box 1).

Box 1 **Key concepts and definitions**

**Cascade interaction**—This is a unidirectional interaction characterized by consumption of another species waste product. In this sense, is a non-reciprocal interaction. A→B.

**Cheater**—An individual obtaining benefits from a public good produced by other(s) that are disproportionately large relative to its own contribution to such good.

**Connectivity**—The flow of molecules and cells across the system.

**Cooperation**—Provision of a benefit available to others at a cost to self.

**Ecological interaction**—Refers to the interactions between producers and consumers of metabolic products in an ecosystem.

**Fitness**—Measure of evolutionary success of an individual in terms of survival and reproduction.

**Frequency dependence**—It refers to evolutionary processes where the fitness of an organism is determined by its relative abundance in the population. It could be positive if fitness increases with frequency or negative when fitness decreases as the organism becomes common in the population.

**Interaction**—Refers to any interaction between the members of an ecosystem, either biotic or abiotic.

**Mass action environment**—We refer to mass action environment as a well-mixed culture where resources are available to all members of the population or community.

**Polymorphism**—Coexistence of two or more clearly different phenotypes, which are in principle the result of genetic differences. It can be understood as biodiversity.

**Reciprocal interaction**—A form of cooperative two-way interaction, or feedback cooperation, where one organism (A) produces resources for others (B) to the detriment of its own fitness and *vice versa* A↔B, the reciprocal benefits should exceed the cost of the production for the interaction to be maintained.

**Selection regime**—Is an experimental design that provides the necessary conditions to allow the survival of only those individuals from a population expressing a particular phenotype or performing a specific function. Only individuals selected by the experimental conditions can reproduce.

**Structured environment** (spatial structure)—This type of environment is best exemplified by either non-mixed cultures, plates or biofilms where resources are localized to the immediate environment of consumers, restricting availability to other members of the population or community. In this environment interactions occur in a localized manner.

For a single species, biological functions tend to persist when the selective benefit of the function or trait is greater than its cost, and are lost if the reverse is true. In multispecies communities, coexistence criteria have a similar structure, building up from component species. For a multispecies community to persist, the biological functions associated with the interspecies interaction must be concomitantly more beneficial to the component species than their respective costs. Therefore when evaluating the effects of ecological interactions and spatial structure, it is necessary to simultaneously consider both the costs and benefits of the traits of interest and what affects this ratio. We evaluate two main categories of interactions, cascade (non-reciprocal) and reciprocal. The first is a strategy dependent mainly on large-scale community connectivity with broad availability of resources and other molecules, whereas reciprocal interactions involve cooperative feedback and local connectivity.

### Cascade interactions

Cascade type interactions are non-reciprocal, in which metabolites produced by one species or genotype affect the growth of other species. These interactions are characterized by niche partitioning and unidirectional cross-feeding, also called incidental cross-feeding (*sensu* Bull and Harcombe, 2009), where one species uses another's waste as a resource. This is because resource specialist genotypes are generally competitively superior to generalist genotypes when there is an abundance of resources. Surprisingly, this is true even for resources produced by the microbes themselves (Friesen et al., 2004) and adaptation to growth on an exogenously supplied primary nutrient causes reductions in the ability to grow on metabolites. Waste metabolites are excreted to the environment, which are then available for use by other strains that subsequently specialize for growth using these secondary metabolites (Rosenzweig et al., 1994). The waste metabolite resources are inherently associated spatially with the producing bacteria, and as a consequence the biological significance of cascade interactions crucially depends upon the movement of nutrients away from the producing microbes.

**Figure 1:**
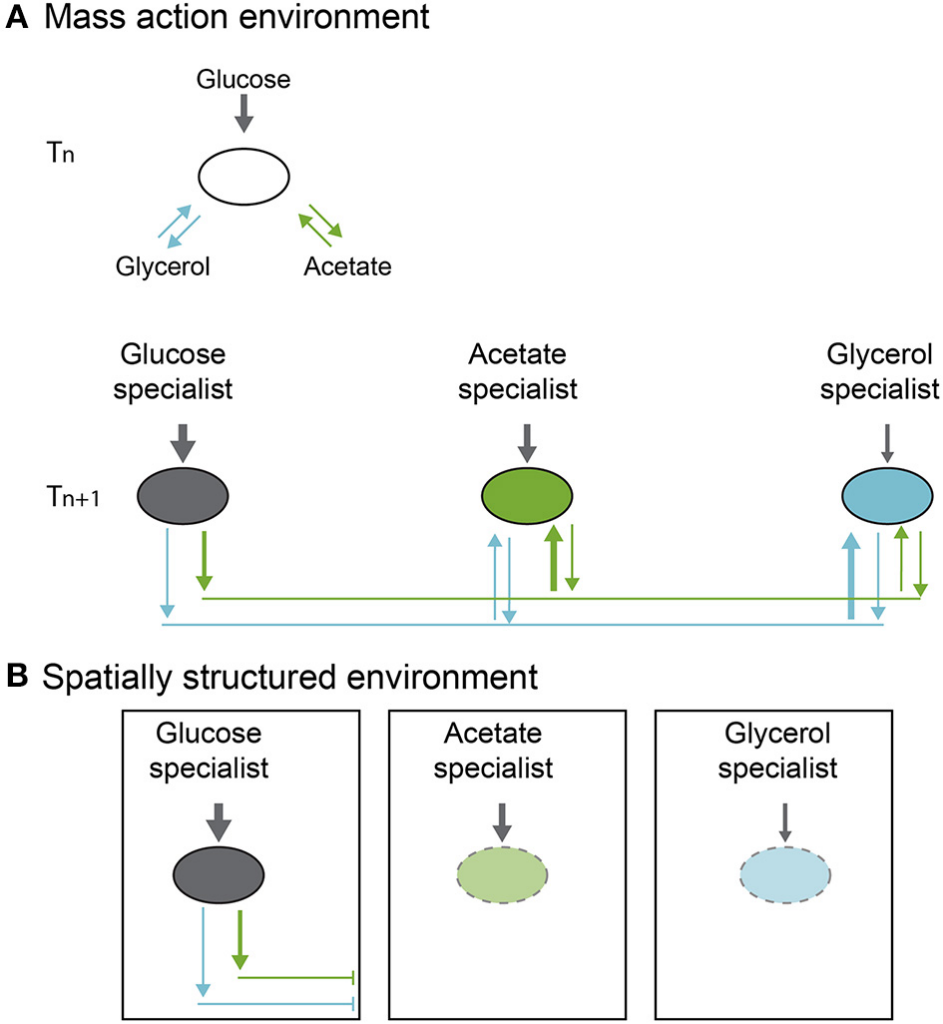
**Polymorphism evolution in a cross-feeding cascade system. (A)** In a mass action environment, polymorphism is maintained through free flow of metabolites (arrows). **(B)** On a spatially structured environment, connectivity is limited and consequently flow of metabolites or resources is reduced affecting polymorphism stability (figure based on the results of Rosenzweig et al., 1994; Saxer et al., 2009).

Cascade interactions are well known in bacteria (Helling et al., 1987; Rosenzweig et al., 1994; Turner et al., 1996; Treves et al., 1998; Rozen and Lenski, 2000). Probably the best-studied example involves the appearance of polymorphisms during evolution of *E. coli* in glucose minimal medium, which was first reported by Helling et al. in 1987. The work by Helling describes the emergence of stable genetic variants in *E. coli* populations during evolution in a chemostat with a single carbon resource (glucose). *E. coli* variants were first identified on Tryptone Agar (TA) plates, where different colony sizes were observed (Helling et al., 1987). Based on chemostat model of coexistence, only a single strain can persist when there is a single limiting resource in a temporally constant environment, the strain that can replace itself at the chemostat washout rate at the lowest resource concentration (Hansen and Hubbell, 1980; Tilman, 1982, 1988). Thus, it was not immediately clear how an *E. coli* multiple genetic variants could arise and be maintained. In later experiments Rosenzweig et al. (1994) demonstrated that these genetic polymorphisms were maintained by cross-feeding interactions, where a glucose specialist consumes only glucose and produces, as byproducts, glycerol and acetate, which in turn, are consumed by two other genotypes (acetate and glycerol specialists) (Figure 1A).

Insights on the origin and maintenance of these polymorphisms have been made by working with polymorphic *E. coli* types from a long-term evolution experiment (Rozen and Lenski, 2000). The populations were derived from replicate cultures, starting from single identical clones in liquid glucose-limited medium, grown under identical conditions of temperature and shaking, and transferred each day to fresh media. In one of the replicate populations of this evolution experiment, a polymorphism evolved after 20,000 generations of selection. Two morphotypes, one with small colonies (*S*) and one with large ones (*L*) were identified. After isolation of the colony types, competition experiments were performed and showed that each type grew better when rare, and coexistence was possible after frequency fluctuations toward an equilibrium point. This polymorphism was maintained even though the *L* type had much higher maximum growth rate in the culture medium and was, therefore, expected to exclude *S* by competition. However, the *S* clone had two advantages that allowed it to invade and coexist with the *L* morphotype. The first advantage was an increasing death rate of *L* when *S* is more abundant, and second, both, *L* and *S* excrete to the medium metabolites that promote *S* growth (metabolite cascade). Coexistence is therefore maintained through frequency dependence and cascade type interactions (Figure 1A).

Following up on the ecological causes for the persistence of polymorphisms, Saxer et al. (2009) showed that when there is no nutrient limitation and spatial structure is added to these polymorphic *E. coli* populations, diversity is lost (Figure 1B). They first selected populations under culture conditions similar to those in Rozen and Lenski's work (2000), but at higher nutrient concentrations to promote production of metabolites and cross-feeding. After obtaining different specialists with differing colony morphotypes (*L* and *S*), the culture conditions were modified to a spatially structured environment, the same culture media with added solidifying agent (non-nutritive agar). Propagation of the cultures was performed by extracting a plug from the agar, dispersing the bacteria in saline solution and transferring them onto fresh medium. Environmental structure in the agar plates impeded dispersal of metabolic waste products, disadvantaging the non-glucose specialists and disrupting coexistence by breaking the stabilizing ecological interaction. Diversity plummeted 50% over 7 days (Figure 1B).

Cheating is not possible in cascade interactions, since there is no requirement for cooperation, the only requirement is resource availability to the specialists. Thus, as long as there is diffusion of resources, coexistence will be maintained. It should be noted, however, that different trade-offs in resource utilization must also exist among populations, since competition for the same resource represents a severe constraint for stability or long-term coexistence (see simulation in Box 2).

Box 2 **Dynamic comparison between two types of consortia in different conditions.**

This review postulates that spatial structure and positive ecological feedbacks promote stability in microbial consortia, especially while looking for cooperative interactions and cheater control. To illustrate this, a couple of toy models corresponding to systems with and without positive feedback (cooperative and cascade-like, respectively) were mathematically specified, which allowed us to analyze their dynamics in different scenarios. In these models, X represents a substrate that is consumed by A, which in turn produces food for B. In the cooperative model (left-hand side), B is able to produce X, thus closing the positive feedback. In the cascade model (right-hand side), X is always available, which could correspond to an external input along the simulations. Note that in the case of the feedback system, C represents a cheater that feeds on X but does not contribute to the persistence of A and B. In the cascade model, C would correspond to a competitor that consumes the same substrate that A consumes.

Both models were initialized exactly with the same amount of A, B, and X. Also, in both cases the consortia were assumed to be on a 2D medium with modular spatial structure, as shown in the figure. These basic models were simulated in scenarios that combined the following conditions: (i) the components of the system remain at the place where they were initially set, (ii) the components of the system can move or are moved in a diffusive manner, therefore homogenizing the spatial arrangement, (iii) the elements denoted as C in the graphs are never present, and (iv) the elements denoted as C arise in each spatial module with a 0.5 probability.

Although these simulations do not explore the possible conditions and parameters exhaustively, they illustrate the dynamics that each of the consortia could exhibit under different conditions. The plots in the figure show how the absolute population sizes changes in time in each of tested scenarios. In summary, these toy-model simulations show that in cooperative systems cheaters can be controlled and the whole population can persist if the spatial structure remains modular, this is, if the members of the community remain where they started or if the environment is not being mixed. A similar situation is observed for the cascade system, but with competitors arising in the community instead of cheaters. However, in the cascade system, the well-mixed condition does not lead to the collapse of the system in the presence of competitors, but to an oscillatory state with even higher average population sizes. It is worth noting that while the cascade system does not collapse in any of the scenarios, its persistence depends on the external X input, while the cooperative system is in principle able to self-sustain once it is “set on.”

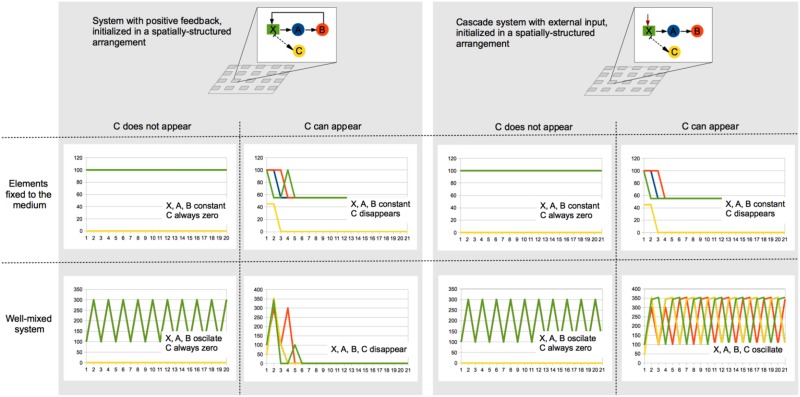

### Reciprocal interactions

Reciprocal interactions involve codependency. Individual organisms produce resources that facilitate the growth of others, and these other organisms provide ‘reciprocal’ resources to the first group of individuals. Frequently, both sets of resources are produced at a short time scale fitness cost to the organisms producing them (i.e., cooperative cross-feeding; Bull and Harcombe, 2009), but with a gain at intermediate time scale (by reciprocation). This type of interaction can readily breakdown, due to the evolution of “cheater” individuals that receive the benefits of the facilitation without contributing (Nowak, 2006). Typically cheaters do not produce resources, but still exploit the resources produced by partner organisms. Most studies that have investigated cooperative behavior invariably consider the evolution of cheater individuals in the populations, and the consequent destabilization and eventual crash of the cooperative system. Depending on the type of cooperative system and environmental spatial structure, this potential meltdown can be overcome or delayed by one or more of three cheater control strategies (Travisano and Velicer, 2004). There can be mechanisms enabling individuals to differentially reward cooperative instead of non-cooperative partners via targeted benefit or targeted punishment limits (Travisano and Velicer, 2004; Momeni et al., 2013), The physical structure of the environment can limit the spread of cheating genotypes, such as spatial structure. A third strategy are physiological and developmental mechanisms that essentially structure the environment temporally, again limiting the spread of cheater genotypes that would disrupt cooperation (Furusawa and Kaneko, 2002; Winther, 2005; Hammerschmidt et al., 2014).

Cancer is an example of cheating in a multicellular system that provides some insight in understanding how cheaters can be overcome in microbial systems. If cheating (cancer) cells remain localized forming a benign tumor, cheating has only modest effects, as the effects are localized. However, if the cheating cells spread (metastasize), then the individual typically dies, as the deleterious effects of cheaters are global. Similarly, in communities, diversity can be maintained if cheaters cannot spread through so that beneficial reciprocal interactions persist. Community spatial structure provides a route for sustained reciprocity as the benefits of resource production are localized to the individuals bearing the cost of resource production. Spatially structured environments provide a mechanism that directs benefits to cooperating individuals (Griffin et al., 2004; Sachs et al., 2004) facilitating the direction of such benefits by localizing interactions (Harcombe, 2009). Indeed, it has been shown that for certain models of cooperation, the organization of communities in subsets of closely interacting individuals can lead to the stabilization of cooperation. Such an organization may certainly correspond to spatial structure, but also to temporal isolation of subsets of individuals or to the non-random architecture of ecological networks (Nowak, 2006).

**Figure 2:**
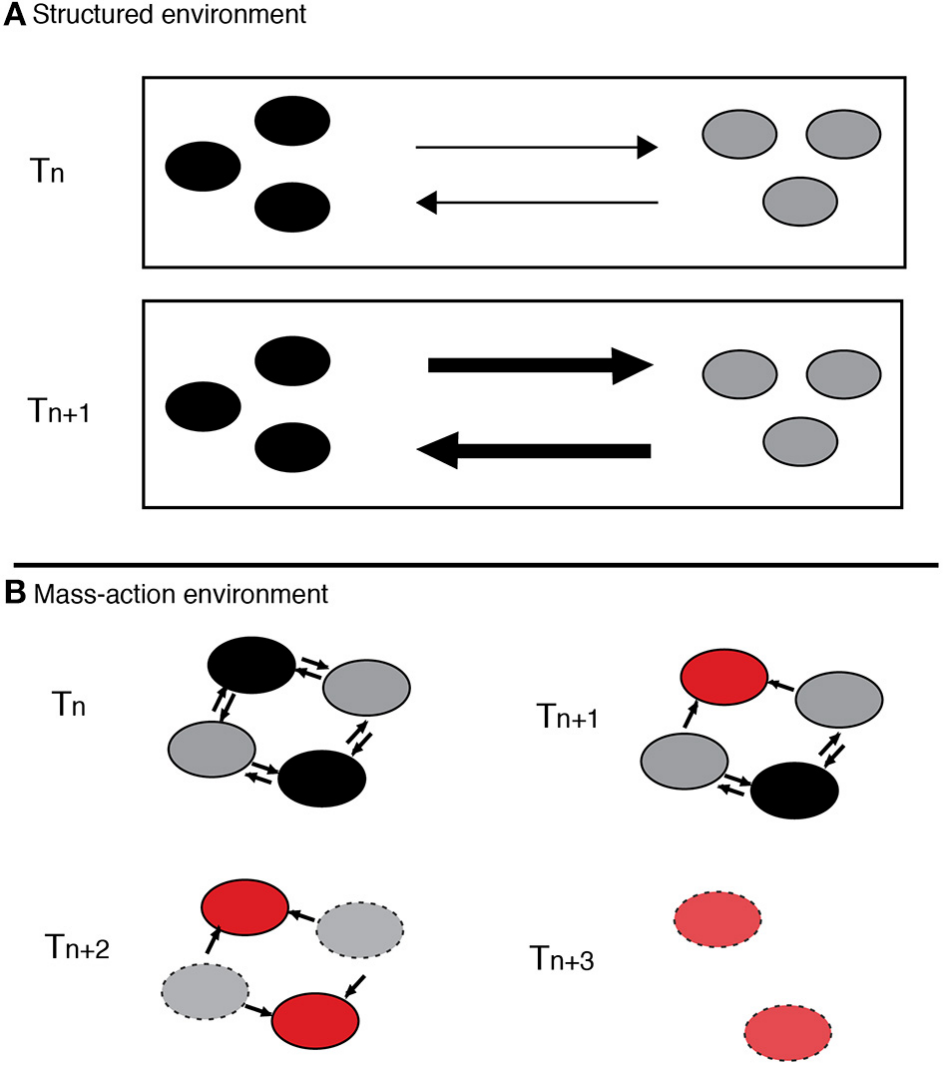
**Polymorphism evolution in a reciprocal interactions system. (A)** On a spatially structured environment diversity is maintained through cooperative interaction. With time this interaction is selectively reinforced, and as a result each species' cooperativeness is increased. Arrows represent metabolites flux. **(B)** On a mass-action environment, however, there is more connectivity, all cells obtain the benefits, even if they do not pay the cost. Therefore, over time, cheaters arise and spread destabilizing the system.

Recently, Harcombe et al. (Bull and Harcombe, 2009; Harcombe, 2009, 2010) have shown that cooperation and the associated diversity can evolve and be maintained in laboratory conditions if there is preexisting reciprocal feedback for cooperation, and if reciprocal interactions are selectively superior to non-reciprocal (cascade) interactions for individual microbes. The experiments demonstrate that it is possible to create and maintain cooperation, if there is initially a low cost production of a resource that a second party can utilize and reciprocally benefit the producer to have more of the waste product. However, this stability is only sustainable in a spatially structured environment where the competitive benefits to cheaters are severely limited. If the environment loses spatial structure, the benefits are available to all, even non-cooperators, making possible the rise of cheaters and the breakdown of cooperation. Harcombe's experiments are direct evidence that spatial structure of the environment is a determinant in directing the benefits to cooperating individuals and localizing interactions (Figure 2), which facilitates stability of a system where ecological reciprocation exists.

These examples of reciprocal interactions show the primary importance of cheating control when looking for stable and long-term coexistence of microbial populations. The examples also illustrate that engineering a spatially structured selective environment can assist in maintaining reciprocal interactions. Both resources (communication signals, nutrients, etc.) and interactions are locally contained and community meltdowns due to invasions by cheaters only occurs at a local scale (see toy-model simulation in Box 2).

## Ecological perspectives of coexistence of engineered consortia

In the previous section we used key examples from experimental evolution of microbial populations to present evidence demonstrating the importance of ecological interactions and spatial structure on long-term system stability in terms of polymorphism maintenance or genetic variants coexistence. In this section we give an ecological and evolutionary perspective for some of the challenges in the field of synthetic biology. Such challenges frequently occur because microbial communities or engineered consortia are visualized as fixed circuits, rarely considering the importance of ecological interactions and adaptation.

We review recent publications on engineered microbial consortia to show that ecological and evolutionary complexity of these systems can easily reach levels where their evolutionary fate becomes hard to predict, thus control of the functionality of the system in the long run can be problematic. Kwok (2010) has attributed the general uncertainty on the behavior of engineered consortia to their complexity in terms of the number of components, potential incompatibilities among them and the impossibility of maintaining all parameters, components and conditions without variation. We argue that, at least in part, design of microbial communities is limited by the lack of an ecological and evolutionary perspective. Adding insights from ecology to the current approach will not overcome all the challenges, however it can help improving performance, stability and predictability of the systems. We focus our discussion of the reviewed examples in three aspects of their design: type of ecological interaction (reciprocal or non-reciprocal), physical structure (mass-action or spatially structured) and observed or predicted outcomes in terms of evolutionary stability of microbial consortia.

**Figure 3:**
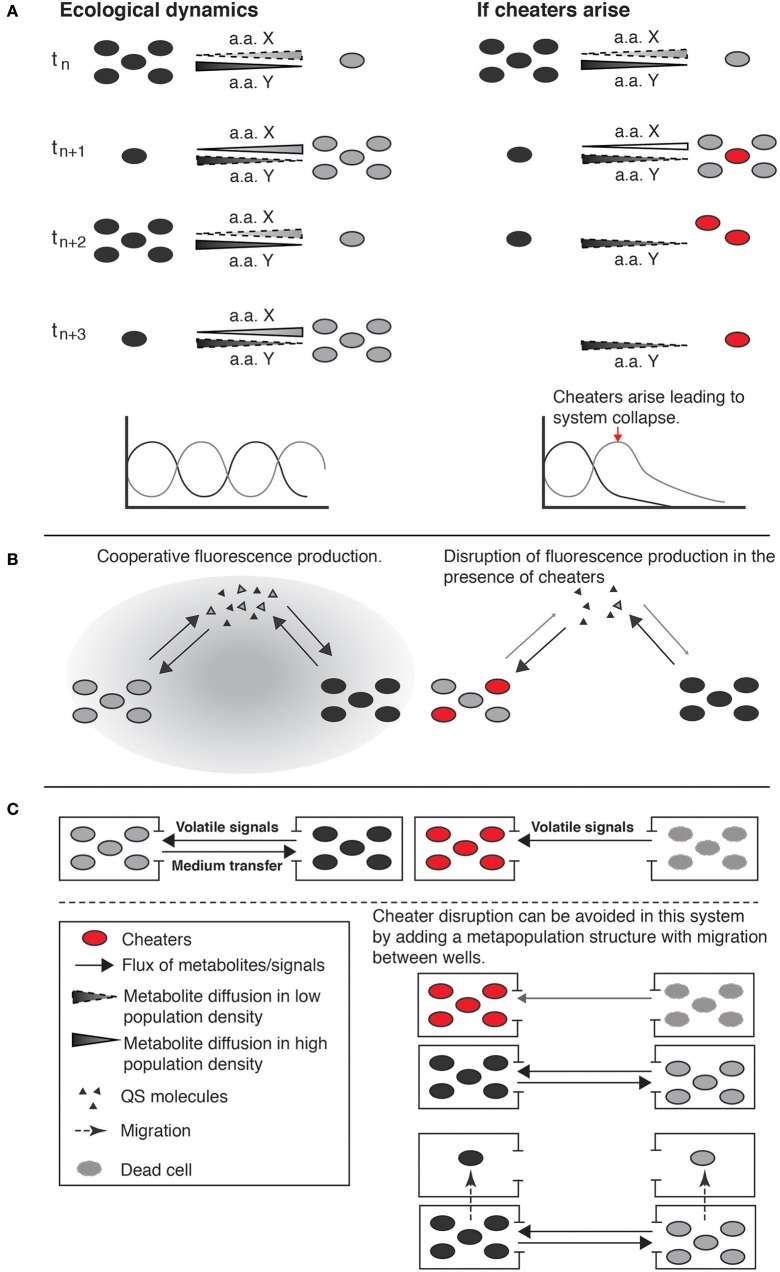
**Ecological dynamics and potential evolutionary outcomes of engineered cooperative consortia**. In the left column are stable short-term dynamics; the right column, illustrates long-term outcomes if cheaters arose by mutation. **(A)** Two populations with strict interdependence by the production of essential aminoacids (one strain produces the aminoacid that the other cannot and *vice versa*). The release of the specific metabolic product is associated with near cell death of the producer and the decline of population density, producing population oscillatory dynamics. Over longer periods of time, cheaters can appear in the population, obtaining the benefits without paying the associated costs, and in the absence of spatial structure cheaters can spread leading to the collapse of the system (example from Shou et al., 2007). **(B)** A microbial consortium produces fluorescence as a cooperative trait regulated by a mechanism of consensus quorum sensing (QS). The signal molecules of this QS mechanism act as positive feedback between the two populations of cells for the production of both fluorescence and more signal molecules. The ecological role of each population is not described but cheaters can arise and disrupt the system, impeding fluorescence production (example from Brenner et al., 2007). **(C)** Reciprocal interaction circuit where two populations of cells are maintained in culture. One of the populations provides essential components to the other through volatile signals or direct transfer of medium, this system is thus open to the evolution of cheaters and collapse of the system. System collapse could potentially be prevented by including a redundancy component and local extinction and migration (metapopulation structure) via the replacement of populations in which cheaters appear with populations in which cheaters are absent (example from Weber et al., 2007).

Work by Shou et al. (2007) is a good example of how engineered consortia can be better understood and designed, if simple ecological and evolutionary principles are taken into consideration. The system was designed taking into account ecological feedbacks (reciprocal interaction), consisting of two engineered yeast genotypes that were cultured with no explicit reference to any spatial structure in liquid media. Each genotype was auxotrophic for a specific amino acid that was overproduced by the other genotype. This strict dependence on the production of essential amino acids generated frequency-dependent selection that stabilized the community. In this system, a genotype increases in frequency when it is rare due to the abundance of its growth limiting essential amino acid. In contrast, the more common genotype decreases in frequency because of the scarcity of its growth limiting amino acid, which is produced by the rare genotype (Figure 3A).

The stabilizing ecological interactions could be lost, however, by the evolution of “cheaters” that do not contribute amino acids to the culture media and gain a growth advantage by using the resources for their own growth and reproduction. Waite and Shou (2012) subsequently showed that the engineered system could be maintained, despite the appearance of cheaters, “… if during adaptation to an environment, the fitness gain of cooperators exceeds that of cheaters by at least the fitness cost of cooperation.” Moreover, a recent follow up on the Shou group system (Momeni et al., 2013) explicitly investigates the role of spatial structure in the successful coexistence of cooperative consortia. What the authors found experimentally, and with computer simulations, is that given certain viscosity of the media (structured environment) and the genetically engineered cooperative behavior, a spatial self-organization favors cooperation over cheating since, the difference in fitness between cooperators and cheaters on the local partners during colony growth into available space drives assortment and automatically grants cooperators instead of cheaters more access to cooperative partners, thus disfavoring cheaters and ensuring partner fidelity (Momeni et al., 2013).

Brenner et al. (2007) similarly engineered a microbial consortium, but with more limited stability. The system involved positive feedbacks, in this case producing a fluorescence compound with a consensus quorum sensing (QS) control mechanism, and tested the cultures in liquid and solid phase (mass-action and spatially structured) (Figure 3B). While the QS mechanism acted as positive feedback for the production of QS molecules and thereby of fluorescence, there was no ecological feedback (cost/benefit of production) maintaining stability as with Shou et al. (2007). Thus this system could potentially be destabilized by loss of function mutants, involving reduced expression of QS molecules, reduced sensitivity to QS control, or inability to produce the fluorescing compounds. These examples illustrate how consideration of ecological and evolutionary dynamics could counteract problems in understanding and engineering consortia that involve long-term instability and unpredictability.

**Figure 4:**
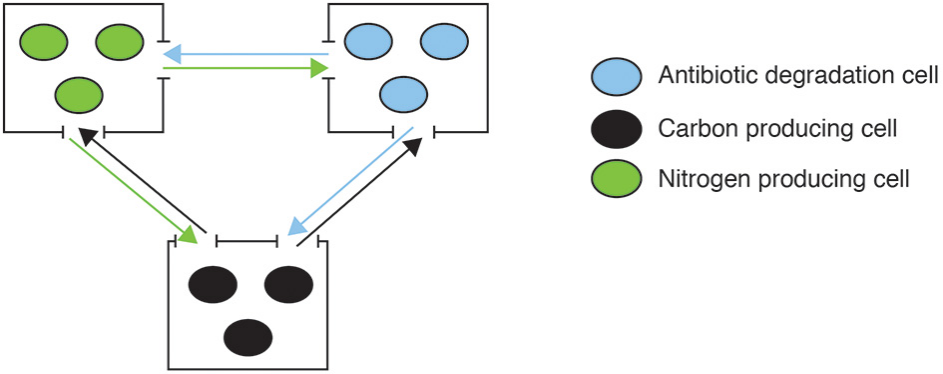
**An engineered consortia with three different soil bacterial populations each contributing with essential resources to the system**. Each of the populations produces different resources (N, C, and antibiotic degradation enzymes) that are necessary and not produced by the others. All populations are kept in independent culture wells, imposing structure with constant connectivity through resources flow (example from Kim et al., 2008).

In contrast, Kim et al. (2008) present the case of an engineered consortium that initially involved spatial structure to maintain stability. It consisted of three different bacteria each contributing with essential resources to others, establishing reciprocal interactions (Figure 4). Each population was grown in individual culture wells, imposing structure with connectivity maintained by chemical communication flow. The system was stably maintained, in part because spatial structure provides the means for cheating control by allowing asymmetric fitness effects of cooperators and cheaters on coexisting populations during colony growth (Momeni et al., 2013), and maintaining disruptive effects of cheaters local while favoring intraspecific (cooperative versus cheating types within wells) over interspecific competition (between wells) (Amarasekare, 2003). When grown in co-culture (mass action) interspecific competition increases and, due to differences in growth rates, one strain becomes dominant, displacing the others. In this case the community was engineered not only as a circuit but also as an open system highly influenced by external interactions with both the environment and other organisms.

Weber et al. (2007) also involved spatial structure in their engineered biological circuit connecting different cell populations, but with less satisfactory results. They show various types of ecological interactions, but two are particularly relevant for our purposes: first, mutualistic or reciprocal, and second commensal or cascade. In the first case (reciprocal), two populations of cells are maintained in culture conditions (spatially segregated) where one population provides essential components to the other through volatile signals or direct transfer of medium from one population to the other (Figure 3C). If the essential resources produced by one population fail to reach the other, both populations die and the system collapses, in a manner similar to that in Figure 3A, even though the system different components (populations) are spatially segregated. Including our ecological and evolutionary perspective, system collapse could, potentially, be prevented by including component redundancy, replacing populations in which cheaters appear with populations in which cheaters are absent (Kerr et al., 2006). Because the evolution of cheaters is stochastic, depending on mutations, the likelihood of all populations containing cheaters depends upon the number of individuals within a population and the number of populations, both of which are under control of the experimenter.

In the second case (non-reciprocal) in Weber et al. (2007), commensal or cascade interactions also involve two populations spatially segregated, but one of them does not require resources from the other, completely changing the ecological dynamics of the system. In this new arrangement of the system, the main risk for the stability is access to resources, not cheating control, thus spatial structure is counterproductive. However, the original design of the circuit (airborne transport of signals) does not compromise access to resources despite culture of the populations in independent vessels, making the system spatially unstructured in this respect (Figure 3C). This example illustrates the ease with which interactions (and therefore the ecological dynamics of the system) are modified while engineering a system, implying a pressing need to consider ecological aspects to assemble stable and productive microbial consortia.

The examples in this section highlight the importance of interactions and spatial structure on the establishment and maintenance of a stable community or consortium, in other words the incorporation of the ecological context whilst designing the setting to maintain the biological circuit. In summary, if reciprocal cooperation is involved in the design, cheating control strategies should also be included in the design, otherwise cheaters are likely to overtake the system and cause it to collapse. In cascade interactions, cheating will not be a problem, but then the connectivity of the system becomes crucial to allow diffusion of interaction's relevant molecules that may be critical for efficient product output.

## Perspective

The use of microbial consortia to carry out processes in industrial and domestic applications (e.g., pharmaceutical, food, materials, effective microorganisms in agroecosystems) is long established. Mixed microbial consortia can perform complex processes that would prove inefficient or impossible for single species systems. The merits of these systems are realized by the avoidance of trade-offs associated with different steps in a process. Despite their utility and common use, the vast majority of microbial consortia were developed on an *ad hoc* basis, and frequently contain a variety of poorly known genotypes and partially understood processes. Because of this, genetic engineering of microbes and consortia has drawn much attention with the promise of higher control over the microbial systems. However, ecological and evolutionary instability has arisen as a pervasive problem.

Successful cases of synthetic microbial communities have shown the feasibility of engineering genetic circuitries to construct efficient cellular machines through the manipulation of genetic parts. Nonetheless, there have been major difficulties in developing microbial consortia. These systems are typically very complex (Kwok, 2010) and while there has been substantial engineering effort in their development, there has been insufficient inclusion of the necessary biological realism for system analysis and design (Kuhn et al., 2010). With the current engineering approach, the whole organism and ecosystem perspective is frequently missed, efficiency problems are commonly encountered (e.g., difficult to control production due to changes in community composition resulting in low yields and economic losses; (Shong et al., 2012), and the potential for system collapses is always present. Very recently, there have been impressive efforts to incorporate more biology into the circuit design through co-occurrence analyses (Berry and Widder, 2014), and stoichiometry and metabolic network modeling of specific microbial strains (Freilich et al., 2009, 2011; Harcombe et al., 2014). These efforts provide substantial information on the type of interactions that can be established within microbial consortia. We argue that better predictability of consortia behavior will only come from evaluations that take into account the evolutionary dynamics of ecological systems in which cheating, connectivity and costs can be controlled through appropriate selection regimes.

Knowledge achieved through microbial population biology experiments is key for considerable improvements and long-term stability of genetically modified communities. Given this, it is not surprising that some of the great successes in the appropriate use of ecological and evolutionary concepts meet the desired goals of productivity, but also stability, resilience and adaptability. Such successes are frequently accompanied by the emergence and maintenance of cooperative behavior. We foresee fulfillment of the promise of microbial consortia coming from metabolic modeling and engineering approaches, by predicting “successful” interactions between two or more microbial strains through their metabolic capacities (Freilich et al., 2011). To achieve this, we believe that a further step is needed in the design and “engineering” of microbial consortia: explicit application of ecological and evolutionary design principles, involving the specifics of the interactions between microbes (direction, feedbacks, non-reciprocity) and the evolutionary consequences that physical structure of the environment. This ecological and evolutionary view, going beyond gene activity, will be crucial in the assessment of new applications and practices involving microbial consortia.

### Conflict of interest statement

The authors declare that the research was conducted in the absence of any commercial or financial relationships that could be construed as a potential conflict of interest.
